# Biomarkers of neurodegeneration in neural autoantibody-associated psychiatric syndromes: A retrospective cohort study

**DOI:** 10.1016/j.jtauto.2022.100169

**Published:** 2022-10-05

**Authors:** Aaron Levin Juhl, Insa Maria Grenzer, Bianca Teegen, Jens Wiltfang, Dirk Fitzner, Niels Hansen

**Affiliations:** a Department of Psychiatry and Psychotherapy, University Medical Center Göttingen, Von-Siebold-Str. 5, 37075, Göttingen, Germany; b Translational Psychoneuroscience, Department of Psychiatry and Psychotherapy, University Medical Center Göttingen, Von-Siebold-Str. 5, 37075, Göttingen, Germany; c Clinical Immunological Laboratory Prof. Stöcker, Groß Grönau, Germany; d German Center for Neurodegenerative Diseases (DZNE), Von-Siebold-Str. 3a, 37075, Göttingen, Germany; e Neurosciences and Signaling Group, Institute of Biomedicine (iBiMED), Department of Medical Sciences, University of Aveiro, Aveiro, Portugal; f Department of Neurology, University Medical Center Göttingen, Robert-Koch Straße 40, 37075, Göttingen, Germany

**Keywords:** Neurodegeneration, Neural autoantibody, Autoimmunity, Psychiatric syndrome, Neurodegenerative biomarkers

## Abstract

**Background:**

Autoantibody-associated psychiatric syndromes are a novel disease entity that is not fully understood. Several lines of evidence suggest that neurodegenerative processes are involved here. We are investigating whether autoantibody-positive psychiatric syndromes differ from those that are autoantibody-negative in cerebrospinal fluid (CSF) neurodegeneration markers.

**Methods:**

We retrospectively analyzed data from 167 psychiatric patients at the University Medical Center Göttingen from 2017 to 2020. We divided this patient cohort into two, namely antibody-positive and antibody-negative. We compared various clinical features, neurodegeneration markers, and their autoantibody status in CSF and serum. We then compared both cohorts' neurodegeneration markers to a representative Alzheimer cohort. We subdivided the patients into their diverse psychiatric syndromes according to the manual to assess and document psychopathology in psychiatry (the AMDP), and compared the neurodegeneration markers.

**Results:**

Antibody-associated psychiatric syndromes do not appear to reveal significantly greater neurodegeneration than their antibody-negative psychiatric syndromes. 71% of antibody-positive patients fulfilled the criteria for a possible and 22% for a definitive autoimmune encephalitis. Our autoantibody-positive patient cohort's relative risk to develop an possible autoimmune encephalitis was 9%. We also noted that phosphorylated tau protein 181 (ptau 181) did not significantly differ between antibody-associated psychiatric syndromes and our Alzheimer cohort. The psycho-organic syndrome usually exhibits the most prominent neurodegeneration markers, both in antibody-positive and antibody-negative psychiatric patients.

**Discussion:**

We did not find hints for neurodegenerative processes in our antibody-positive versus AD cohort considering total tau or amyloid markers. However, our findings indicate that the neurodegeneration marker ptau181 does not differ significantly between antibody-positive and Alzheimer cohorts, further suggesting axonal neurodegeneration in antibody-positive patients as AD patients have an elevated ptau181. The evidence we uncovered thus suggests that axonal neurodegeneration might affect patients suffering from autoantibody-associated psychiatric syndromes.

## 1 Introduction

In recent years, autoantibodies and elevated neurodegenerative markers have been increasingly detected in association with many neuropsychiatric diseases [[1], [2], [3]]. Various biomarkers in the cerebrospinal fluid (CSF) can indicate neurodegenerative processes. Elevated phosphorylated tau protein 181 (ptau 181) and reduced amyloid-β 42 (Aβ42) are typical biomarker profiles indicating Alzheimer's disease [4], the most common neurodegenerative disease [5]. Autoantibodies do not necessarily indicate pathologies - they are also detected in healthy controls [6]. Autoimmune encephalitis (AE) is a disease frequently associated with neural-autoantibody detection, neuropsychiatric symptoms, and sometimes elevated degeneration markers [2,7,8]. It is a brain inflammation of autoimmune origin [9] that often worsens rapidly and entails the loss of cognitive function [10]. Many types of AE are distinguished depending on the autoantibodies detected, yet their clinical presentation is often quite different [11]. The most common autoantibody associated with AE is anti-NMDAR [12]. In the course of this disease, elevated tau protein in the cerebrospinal fluid is often present, as are other neuropsychiatric symptoms, which usually normalize rapidly with immunosuppressive therapy [2]. However, not every psychiatric disorder revealing evidence of autoantibodies can be diagnosed as AE [13]. It must meet certain strict criteria to be considered AE [9]. It is possible to classify patients presenting autoantibody evidence and psychiatric symptomatology into psychiatric syndromes according to the AMDP (Manual for Assessment and Documentation of Psychopathology in Psychiatry) [14]. This manual describes nine syndromes subdivided according to their clinical presentation [15]. Neurodegenerative biomarkers can also be elevated in psychiatric patients presenting autoantibody evidence who fail to fulfill AE criteria [16]. Although a dysregulated immune system is thought to play a major role in neurodegenerative processes [17], the exact role played by both autoantibodies and elevated neurodegeneration markers remains unclear in many of these diseases [18,19]. Clear answers to these questions would influence further diagnostic and therapeutic options for psychiatric disorders, and thus have direct consequences for the individual patient. The aim of this study is to provide clarity as to whether, and if so, which neurodegeneration biomarkers are significantly increased or decreased in autoantibody-positive tested psychiatric patients compared to autoantibody-negative tested psychiatric patients, and compared to a representative Alzheimer's disease cohort. We also aim to identify whether any of the AMDP system psychiatric syndromes associated with autoantibodies differ from those psychiatric syndromes without antibodies in their neurodegeneration markers.

## 2 Methods

### 2.1 Patients

Our patient group consisted of 167 patients recruited from the Department of Psychiatry and Psychotherapy of the University Medical Center Göttingen from 2016 to 2020. As data of neurodegeneration markers from 44 of the 167 patients was missing, we had to exclude them from the study. For our retrospective studies, data from 123 patients in this patient cohort was relied upon; they underwent examinations for neurodegeneration biomarkers and their neuronal autoantibody status in serum and/or CSF. This 123 patient cohort was divided into “antibody-positive” (Ab-p) and “antibody-negative” (Ab-n) subjects according to their autoantibody status. Not all available antigens were tested in 4 patients (all Ab-n), only their neuronal or paraneoplastic antigens were tested in CSF and/or serum. We also recruited a representative comparison cohort consisting of 27 patients diagnosed with Alzheimer's disease (AD) at different stages and, accordingly, determined their pathological degeneration markers. Data from these 27 comparison patients were obtained from the biobank of the Department of Psychiatry and Psychotherapy, University Medical Center Göttingen.

### 2.2 Statistical analysis

These data were processed and analyzed using IBM SPSS Statistics version 28. First, descriptive statistics were collated for clinical characteristics, such as MRI scans and neurodegenerative biomarkers; we then examined these parameters for significant differences using the Mann-Whitney-U-Test and Chi-Square-Test. Depending on the diagnosis, we divided the patients into diagnostic groups and analyzed them using descriptive statistics to see whether any one diagnostic group would be observed more frequently in Ab-p or Ab-n. Then, the neurodegeneration biomarkers of these various groups were first compared via one-factorial analysis of variance (ANOVA) based on different factors (cohort, group, syndrome> 1 observation). The factor cohort was divided into Ab-p, Ab-n, and AD group of patients. The group factor was divided into Ab-p in serum, Ab-p in serum + CSF, Ab-n and AD. The third factor, syndrome, constitutes the varied syndrome combinations of patients according to the AMDP classification. Because individual observations could not undergo ANOVA, only syndrome combinations >1 observation were included in our analyses. This included a post-hoc LSD to test for significant differences between various factor levels. Because the data in the respective factor levels were often not normally distributed and variance homogeneity was absent, we also ran a Kruskal-Wallis test for all neurodegenerative biomarkers. The patients' psychiatric syndromes were categorized according to the AMDP system based on their psychopathology (data retrieved from the respective physician's letters and other clinical findings).

### 2.3 Neural autoantibodies

The patients' neuronal autoantibodies were determined in the Clinical Immunological Laboratory Prof. Stöcker. In peripheral blood and/or CSF, they detected autoantibodies against surface antigens (α-amino-3-hydroxy-5-methyl-4-isoxazolepropionic acid receptors 1/2 (AMPAR1/2), aquaporin 4, contactin-associated protein-2 (CASPR2), dipeptidyl peptidase-like protein-6 (DPPX), Myelin, Glycine, IgLON5, gamma-aminobutyric acid B1/2 receptor (GABAB1/2R), leucine-rich glioma inactivated protein 1 (LGI1), *N*-methyl-d-aspartate receptor (NMDAR), Potassium Voltage-Gated Channel Subfamily A Member 2 (KCNA2) and against intracellular, mostly paraneoplastic antigens [Amphiphysin, CV2, glutamic acid decarboxylase of kDa65 (GAD65), HuD, Ma1/Ma2, Neurochondrin (NC), Ri, TR, Yo, Titin and zinc finger protein of the cerebellum 4 (Zic4), Recoverin, sry-like high motility group box 1 (SOX1)]. To search for specific neuroglial autoantibodies, we employed BIOCHIP mosaics containing brain tissue and recombinant cells. The BIOCHIP mosaics were built up of human embryonic kidney cells transfected with neuroglial antigens to investigate blood or CSF material. Standard immunofluorescence tests were utilized to identify the aforementioned autoantibodies against intracellular antigens. We also ran immunofluoresecence tests for the autoantibodies against membrane-surface antigens and ion channels described above. Homemade cell-based assays from the Clinical Immunological Laboratory of Prof. Stöcker were used to test for all neural autoantibodies, except for anti-ANNA3 and -myelin antibodies. The neural autoantibodies were all investigated in Prof. Stöcker's Clinical Immunological Laboratory and were assessed semiquantitatively to distinguish between low-, medium-, and high-intensity levels in their biomaterial probes. CSF was analyzed at the Laboratory of Neurochemistry of the University Medical Center Göttingen. These investigations were approved by our local ethics committee and are subject to the actual version of the Declaration of Helsinki.

## 3 Results

### 3.1 Demographics and clinical characteristics of patients

In total, we included 123 patients in these investigations. Twenty-eight of them were assigned to the Ab-b group. The remaining 95 patients were assigned to the Ab-n group. Twenty-seven of 28 presented antibodies in serum, 10 of 123 patients had antibodies in serum and CSF, and 1 of 123 had antibodies only in CSF and not in serum. The patients with antibodies in serum revealed antibodies to Yo (n = 2), Myelin (n = 2), Glycine (n = 2), NMDAR (n = 5), NC and Titin (n = 1), Titin and Yo (n = 1), Titin (n = 1), Amphiphysin (n = 1), GAD65 and Recoverin (n = 1), Recoverin (n = 1), KCNA2 (n = 2), CV2/CRMP5 (n = 2), CASPR2 (n = 2), IgLON5 (n = 1), Neuropil (n = 2), Zic4, SOX1 and Ma1 (n = 1) (Table 1). There was one patient with antibodies in CSF only, but not in serum; those were antibodies to Yo (n = 1). The other antibodies in CSF in patients who also presented detectable antibodies in serum were directed against these antigens: CV2/CRMP5 (n = 1), NC (n = 1), KCNA2 (n = 1), NMDAR (n = 2), CASPR2 (n = 1), Titin (n = 1) and IgLON5 (n = 1), neuropil (unspecific antibodies) (n = 2), and Zic4, SOX1, and Ma1 (n = 1) (Table 1). Overall, our cohort consisted of 55 men and 68 women. Ab-p did not differ significantly in age or sex from Ab-n (Table 1). The Chi-Square-Test and Mann-Whitney-U-Test revealed no significant differences in clinical and laboratory parameters or in diagnoses between Ab-p and Ab-n (Table 2, Table 3). Our AD group had a sample size of n = 27 and was gender-matched. On average, AD-cohort patients were 74 years old (73.78 ± 1.371 years); 16 of the 27 patients were women (59.3%) (Table 2). We also screened our cohort for autoimmune encephalitis. In 20 of 28 patients (71%) we detected a possible autoimmune encephalitis in the autoantibody positive patients (Table 1) and we confirmed a definitive autoimmune encephalitis in 6 of 28 (22%) according to the Graus criteria [9]. Thus, the relative risk to that a autoantibody positive finding culminates in a possible autoimmune encephalitis is 9%.

**Table 1:** Classification of patients with neural autoantibodies.

Patient number	Ab Serum	Ab CSF	Possible AE	Definitive AE
1	CV2/CRMP5	CV2/CRMP5	Present	Present
2	Yo	Not present	Not present	Not present
3	Myelin (unspecific)	Not present	Present	Not present
4	Neurochondrin	Neurochondrin	Not present	Not present
5	Not present	Yo	Present	Present
6	Neuropil	Not present	Present	Not present
7	GlycinR	Not present	Present	Not present
8	GAD65, Recoverin	Not present	Present	Not present
9	Recoverin	-	Not present	Not present
10	KCNA2	KCNA2	Present	Not present
11	Myelin (unspecific)	Not present	Present	Not present
12	Recoverin	-	Present	Not present
13	Zic4	-	Not present	Not present
14	NMDAR	NMDAR	Present	Present
15	Yo	Not present	Present	Not present
16	Glycine	Not present	Not present	Not present
17	Amphiphysin	Not present	Not present	Not present
18	KCNA2	Not present	Not present	Not present
19	CV2	Not present	Present	Not present
20	NMDAR	NMDAR	Present	Present
21	CASPR2	CASPR2	Present	Present
22	Zic4, SOX, Ma1	Zic4, SOX, Ma1	Present	Not present
23	NMDAR	NMDAR	Present	Not present
24	myelin	Not present	Present	Not present
25	Neuropil (unspecific)	Neuropil (unspecific)	Present	Not present
26	Recoverin	-	Present	Not present
27	CASPR2	Not present	Not present	Not present
28	IgLON5	IgLON5	Present	Present

Abbreviations: CASPR2 = contactin-associated protein-2, CV2/CRMP5 = cronveinten 2/Collapsin response mediator protein 5, GAD65 glutamic acid decarboxylase of kDa65, KCNA2 = Potassium Voltage-Gated Channel Subfamily A Member 2, NMDAR = *N*-methyl-d-aspartate receptor, SOX1 = Recoverin, sry-like high motility group box 1, Zic4 = zinc finger protein of the cerebellum 4. Ab PB = autoantibody peripheral blood, Ab CSF = autoantibody cerebrospinal fluid.

**Table 2:** Clinical and laboratory parameter of autoantibody positive and negative patients.

	Parameter	Ab-p	Ab-p (%)	Ab-n	Ab-n (%)	Statistics
Number of patients		28		95		
Gender		m: 14/28 f: 14/28	m: 50 f: 50	m: 41/95 f: 54/95	m: 43,16 w: 56,84	0,522*
Age years		63,93 ± 2,39		61,09 ± 1,4		0,398**
CSF						
	Cell Count (<5 μl)	1,04 ± 0,32		1,53 ± 0,84 (n = 94)		0,559**
	Intrathecal IgG-synthesis	3/28	10,71	9/94	9,57	0,848*
	Blood brain barrier disturbance	5/28	17,86	17/92	18,48	0,941*
	Total protein count	459,36 ± 33,69		420,2 ± 16,09 (n = 91)		0,244**
cMRI	Generalized atrophy	7/25	28	34/84	40,48	0,522*
	Focal atrophy	10/25	40	24/84	28,57	0,550*
	Hippocampal atrophy	1/25	4	5/84	5,95	0,924*
	Vascular lesions	14/25	56	38/84	45,24	0,633*
EEG						
	Temporal focal slowing	7/18	38,89	23/55	41,82	0,885*
	Temporal potentials typical of epilepsy	1/18	5,56	1/55	1,82	0,572*
	Non-temporal focal deceleration	6/18	33,33	22/55	40	0,728*
	Non-temporal potentials typical of epilepsy	0/18	0	1/55	1,82	0,699*

Abbreviations:* = asymptotic two-tailed p-value (Chi-squared test), ** = asymptotic two-tailed p-value (Mann-Whitney *U* test)**,** Ab-n = antibody-negative psychiatric patients**,** Ab-p = antibody-positive psychiatric patients**,** cMRI = cerebral magnetic resonance imaging**,** CSF = cerebrospinal fluid**,** EEG = electroencephalogram**,** f = female**,** m = male.

**Table 3:** Diagnosis of autoantibody positive and autoantibody negative psychiatric patients.

ICD-10	Complete cohort (%)	Ab-p (%)	Ab-n (%)	Statistics*
F00-F09	75/123 (60,98)	21/28 (75)	54/95 (56,84)	0,083
F10-F19	3/123 (2,44)	0/28 (0)	3/95 (3,16)	0,341
F20-F29	7/123 (5,69)	1/28 (3,57)	6/95 (6,32)	0,582
F30-F39	34/123 (27,64)	4/28 (14,29)	30/95 (31,59)	0,072
F40-F49	4/123 (3,25)	2/28 (7,14)	2/95 (2,11)	0,187
F50-F59	0/123 (0)	0/28 (0)	0/95 (0)	-
F60-F69	0/123 (0)	0/28 (0)	0/95 (0)	-

**Abbreviations:** * = asymptotic two-tailed p-value (Chi-squared test), Ab-n = antibody-negative psychiatric patients, Ab-p = antibody-positive psychiatric patients, ICD-10 = 10th revision of the International Statistical Classification of Diseases and Related Health Problems.

### 3.2 Biomarkers of neurodegeneration

#### 3.2.1 Cohort

**Fig. 1:**
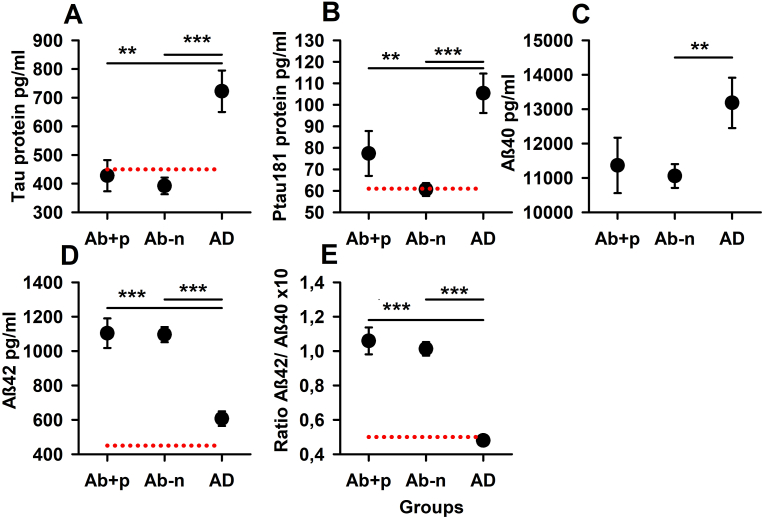
**Differences in neurodegeneration biomarkers between psychiatric patients with and without neural autoantibodies and comparison cohort with AD**. All neurodegeneration biomarkers differ significantly between AD (n = 27) and Ab-n (n = 95) and between AD and Ab-p (n = 28), except for Aβ40. Abbreviations: Ab-p = psychiatric patients with neural autoantibodies, Ab-n = psychiatric patients without neural autoantibodies, AD = comparative cohort with Alzheimer's disease, Tau protein = total tau protein, ptau 181 = phosphorylated tau protein 181, Aβ42 = amyloid-β-42, Aβ40 = amyloid-β-40, Ratio Aβ42/Aβ40 = Ratio amyloid-β-42/amyloid-β-40. All neural autoantibodies were put in one group as subgroups of specific autoantibodies would be too low to make any statement.

The neurodegeneration biomarkers total tau protein (t-tau), ptau 181, amyloid β-40 (Aβ40), Aβ42, and ratio Aβ42/Aβ40 did not differ significantly between Ab-p and Ab-n (Fig. 1). Strikingly, ptau 181 was much higher on average in Ab-p (77.4 ± 10.4 pg/ml) than in Ab-n (60.6 ± 3.01 pg/ml). Thus, ptau 181 in the Ab-p group was pathological on average and well above the cut-off value (ptau 181 cut-off: >61 pg/ml), whereas the Ab-n group revealed inconspicuous ptau 181 values on average. Nevertheless, this difference in the neurodegeneration biomarker ptau 181 was not significant in the Kruskal-Wallis test. Neurodegeneration biomarkers differed consistently between AD and Ab-n in the Kruskal Wallis test (Fig. 1). There was no significant difference in Aβ40 between AD and Ab-p only; otherwise, it was always significant there as well (Fig. 1).

#### 3.2.2 Group

**Fig. 2:**
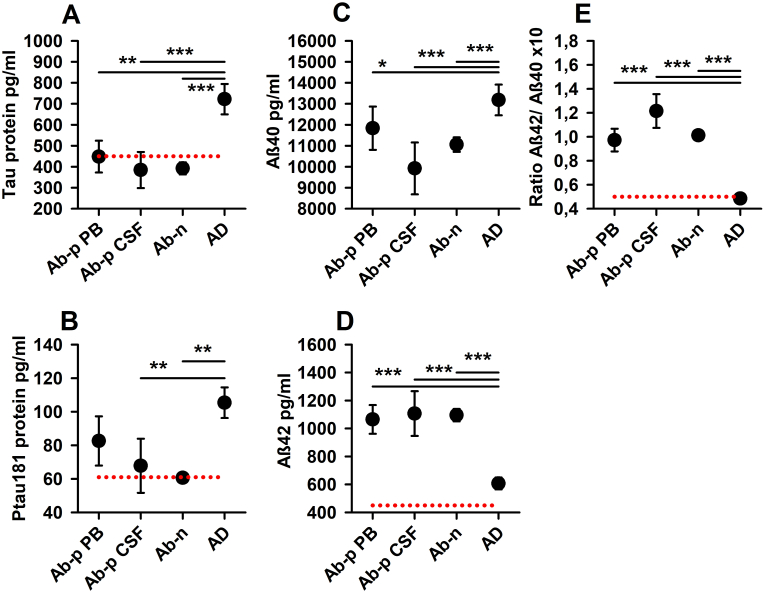
**Differences in neurodegeneration biomarkers between psychiatric patients with neural autoantibodies in peripheral blood, psychiatric patients with neural autoantibodies in peripheral blood and CSF, psychiatric patients without neural autoantibodies and comparison cohort with AD**. All neurodegeneration biomarkers differ significantly between AD (n = 27) and Ab-n (n = 95) and between AD and Ab-p CSF (n = 10). Ptau 181 differs not significantly between AD (n = 27) and Ab-p PB (n = 28). Abbreviations: Ab-p PB = psychiatric patients with neural autoantibodies in peripheral blood (n = 28), Ab-p CSF = psychiatric patients with neural autoantibodies in peripheral blood and CSF (n = 10), Ab-n = psychiatric patients without neural autoantibodies (n = 95), AD = comparative cohort with AD (n = 27), Tau protein = total tau protein, ptau 181 = phosphorylated tau protein 181, Aβ42 = amyloid-β-42, Aβ40 = amyloid-β-40, Ratio Aβ42/Aβ40 = Ratio amyloid-β-42/amyloid-β-40.

The Ab-p in serum, Ab-p in serum + CSF, and Ab-n groups did not differ significantly from each other (Fig. 2). AD differed significantly from Ab-p in serum + CSF and Ab-n in all degeneration markers (Fig. 2). AD did not differ from the Ab-p-in-serum group in the ptau 181 neurodegeneration biomarker, but AD did differ significantly in their remaining neurodegeneration biomarkers (Fig. 2).

#### 3.2.3 Syndrome combination

**Fig. 3:**
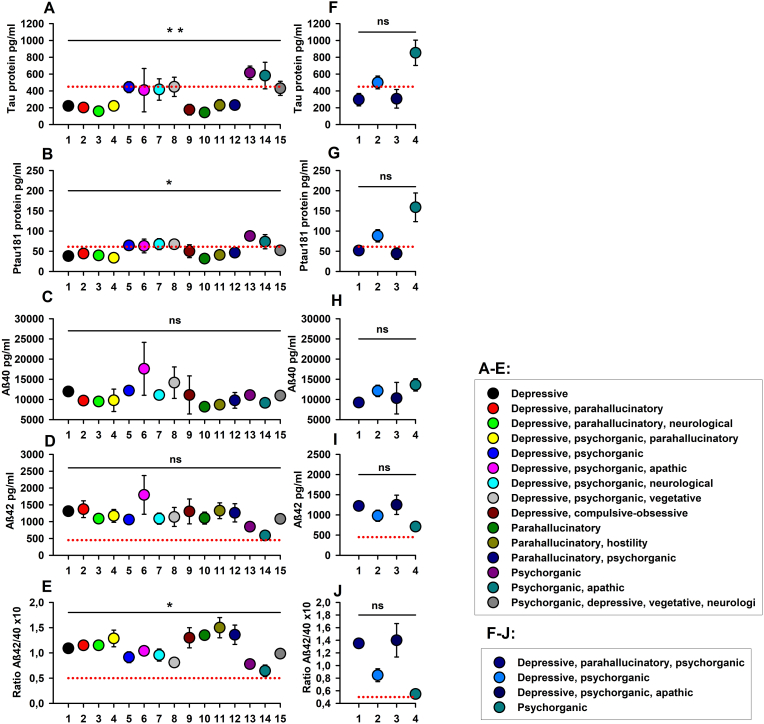
**Differences in neurodegeneration biomarkers between syndromes and syndrome combinations – AMDP system**. The psycho-organic syndrome or a syndrome coinciding with psychoorganic syndrome usually exhibited the most prominent neurodegeneration biomarkers in both autoantibody-positive and autoantibody-negative patients. Abbreviations: ns = not significant A–E: AMDP-Syndromes from psychiatric patients without neural autoantibodies, F–J: AMDP-Syndromes from psychiatric patients with neural autoantibodies, Tau protein = total tau protein, ptau 181 = phosphorylated tau protein 181, Aβ42 = amyloid-β-42, Aβ40 = amyloid-β-40, Ratio Aβ42/Aβ40 = Ratio amyloid-β-42/amyloid-β-40. Antibody-positive patients (Ab-p): depressive, parahallucinatory, psychoorganic syndrome (t-tau: n = 2, ptau181: n = 2, Aβ40: n = 2, Aβ42: n = 2, Aβ42/40: n = 2); depressive, psychoorganic syndrome (t-tau: n = 9, ptau181: n = 9, Aβ40: n = 10, Aβ42: n = 10, Aβ42/40: n = 10); depressive, psychoorganic, apathic snyndrome (t-tau: n = 3, ptau181: n = 3, Aβ40: n = 3, Aβ42: n = 3, Aβ42/40: n = 3); psychoorganic syndrome (t-tau: n = 4, ptau181: n = 4, Aβ40: n = 4, Aβ42: n = 4, Aβ42/40: n = 4). Antibody-negative patients (Ab-n): depressive syndrome (t-tau: n = 5, ptau181: n = 5, Aβ40: n = 5 Aβ42: n = 5, Aβ42/40: n = 5); depressive, parahallucinatory syndrome (t-tau: n = 3, ptau181: n = 3, Aβ40: n = 2, Aβ42: n = 3, Aβ42/40: n = 3); depressive, parahallucinatory, neurological syndrome (t-tau: n = 2, ptau181: n = 2, Aβ40: n = 2, Aβ42: n = 2, Aβ42/40: n = 2); depressive, psychoorganic, parahallucinatory syndrome (t-tau: n = 2, ptau181: n = 2, Aβ40: n = 3, Aβ42: n = 3, Aβ42/40: n = 3); depressive, psychoorganic syndrome (t-tau: n = 21, ptau181: n = 21, Aβ40: n = 19, Aβ42: n = 21, Aβ42/40: n = 21); depressive, psychoorganic, apathic syndrome (t-tau: n = 2, ptau181: n = 2, Aβ40: n = 2, Aβ42: n = 2, Aβ42/40: n = 2); depressive, psychoorganic, neurological syndrome (t-tau: n = 9, ptau181: n = 9, Aβ40: n = 9, Aβ42: n = 9, Aβ42/40: n = 9); depressive, psychoorganic, vegetative syndrome (t-tau: n = 2, ptau181: n = 2, Aβ40: n = 2, Aβ42: n = 2, Aβ42/40: n = 2); depressive, compulsive-obsessive syndrome (t-tau: n = 2, ptau181: n = 2, Aβ40: n = 2, Aβ42: n = 2, Aβ42/40: n = 2); parahallucinatory syndrome (t-tau: n = 2, ptau181: n = 2, Aβ40: n = 2, Aβ42: n = 2, Aβ42/40: n = 2); parahallucinatory, hostility syndrome (t-tau: n = 2, ptau181: n = 2, Aβ40: n = 2, Aβ42: n = 2, Aβ42/40: n = 2); parahallucinatory, psychoorganic syndrome (t-tau: n = 5, ptau181: n = 5, Aβ40: n = 5, Aβ42: n = 5, Aβ42/40: n = 5); psychoorganic syndrome (t-tau: n = 16, ptau181: n = 16, Aβ40: n = 16, Aβ42: n = 16, Aβ42/40: n = 16); psychoorganic, apathic syndrome (t-tau: n = 2, ptau181: n = 2, Aβ40: n = 2, Aβ42: n = 2, Aβ42/40: n = 2); psychoorganic, depressive, vegetative, neurological syndrome (t-tau: n = 2, ptau181: n = 2, Aβ40: n = 2, Aβ42: n = 2, Aβ42/40: n = 2).

Ab-p's neurodegeneration biomarkers did not differ significantly between syndrome combinations (Fig. 3). Patients with psychoorganic syndrome (Psyorg) had on average the highest t-tau (853.3 ± 150.7 pg/ml), ptau 181 (158.8 ± 35.4 pg/ml) and Aβ40 (13597.3 ± 1519.5 pg/ml) values among patients with Ab-p. The ratio Aβ42/40 was the lowest in the Ab-p group's Psyorg patients (0.55 ± 0.07). Aβ42 was highest among Ab-p patients in the syndrome combination “Depres; Psyorg; Apa” (syndrome combination: depressive, psychoorganic and apathic syndrome) (1250.7 ± 238.5 pg/ml). Aβ42 was lowest in the Ab-p group in conjunction with Psyorg syndrome (710 ± 30.9 pg/ml). Ab-n patients differed significantly between syndrome combinations in the neurodegeneration biomarkers t-tau, ptau 181, and in the ratio Aβ42/40 (Fig. 3). Among patients in the Ab-n group, on average, those with a psyorg revealed the highest levels of t-tau (615.4 ± 79.9 pg/ml) and ptau 181 (87.5 ± 7.4 pg/ml). Aβ40 was highest among Ab-n patients in the syndrome combination “Depres; Psyorg; Apa” (17,575 ± 6557 pg/ml), just as Aβ42 was highest in this syndrome combination (1795.5 ± 575.5 pg/ml). The ratio Aβ42/40 was lowest in the syndrome combination “Psyorg; Apa” (syndrome combination: psychoorganic and apathic syndrome) (0.65 ± 0.12). The interaction between syndrome and patient groups (Ab-p, Ab-n, AD) revealed no significant differences in neurodegeneration biomarkers in a two-factorial ANOVA.

## 4 Discussion

Our studies show that many different autoantibodies are associated with psychiatric syndromes that in turn can reveal various symptoms, courses, and possibly prognoses. These observations are consistent with other studies' [[20], [21], [22], [23]]. In addition, there appears to be no significant neurodegeneration difference between psychiatric syndromes with and without autoantibodies. This finding contradicts other studies that reported measurable neurodegeneration in autoantibody-associated psychiatric disorders [1,8,16]. Autoantibody-associated psychiatric disorders like AE can be associated with measurable neurodegeneration [2]. In patients presenting various neuropsychiatric disorders, autoantibodies are frequently detected in combination with elevated neurodegeneration biomarkers, whether anti-IgLON5 in combination with elevated ptau 181 [24] or elevated ptau 181 and t-tau coinciding with anti-recoverin [16]. Currently, however, the role of autoantibodies and elevated neurodegeneration biomarkers in these psychiatric patients is largely unknown [18,19]. Neuronal autoantibodies are sometimes associated with neurodegenerative pathologies; an atypical disease course or atypical clinical symptoms may be indicative of such a constellation [3]. There also appear to be varying degrees of neurodegeneration depending on the antibodies detected, for example, there is research evidence that autoantibodies such as anti-Hu, anti-Ma2, and anti-GAD are associated with severe neuronal damage, whereas other autoantibodies are associated with less neuronal damage [25]. AD's neurodegeneration biomarkers differed significantly from the neurodegeneration biomarkers in both Ab-p and Ab-n in the vast majority of patients. This finding confirms studies that reported AD associated with significantly decreased Aβ42 and a lower ratio Aβ42/40 compared to patients suffering from autoantibody-associated cognitive dysfunction [26]. Tau enables us to distinguish between AD and autoantibody-associated dementia, since this biomarker is significantly elevated in AD patients [27]. Another of our studies' findings is that the Ab-p group's neurodegeneration biomarkers did not differ significantly between syndromes. It seems that in psychiatric patients in whom autoantibodies are detected, the presence of neurodegenerative processes cannot be inferred from their psychiatric syndrome. The syndrome that showed the most striking neurodegeneration biomarkers within the Ab-p group was Psyorg. This is not surprising, since the symptoms constituting a Psyorg largely overlap with AD's [28], where a neurodegenerative pathogenesis is known to exist [4]. In the Ab-n group, patients with the different syndromes differed significantly in the neurodegeneration biomarkers t-tau, p-tau 181 and the ratio Aβ42/40. It seems that among those psychiatric patients in whom no autoantibodies were detected, the syndrome factor made a significant difference. Psyorg was also associated with the most prominent neurodegeneration markers in Ab-n patients. Here, the rationale resembles that in the Ab-p group patients.

### 4.1 Limitations

Our AD comparison cohort was not age-matched with our psychiatric patient population. This is because AD is usually a disease of the elderly [29,30], whereas our psychiatric patient cohort represents a cross-section of diverse age groups. The AMDP system is one characterized by graduations. However, the records do not yield enough data to make such firm statements. Making a graduation as the AMDP recommends would require several days of each patient's personal experience [28]. There were also cases in which only antibodies in serum were assessed, not in the CSF. The lack of a healthy control group that had undergone lumbar puncture is another study limitation. All patients in our cohort and some in the control group were examined with a bias, because we could not subject any patient be to a lumbar puncture without an indication. This is an unavoidable limitation, since lumbar puncture entails various risks and performing it without any medical indication would be negligent [31]. Immunoblots have the risk of false positivity for paraneoplastic autoantibodies, as a study by Dechelotte showed [32]. To circumvent this disadvantage, we used cell-based assays to detect a variety of neural autoantibodies. Specific autoantibodies associated with psychiatric disorders are likely irrelevant, ie, (1) aquaporin-4 autoantibodies and (2) titin autoantibodies, which are often associated with (1) neuromyelitis optica and (2) myasthenia gravis. Another study limitation is that we put all autoantibody-positive patients into a group because studying specific neuronal autoantibodies would be pointless because we had too few subjects.

## 5 Conclusion

Our results indicate that there does not seem to be significantly more severe neurodegeneration in autoantibody-associated psychiatric syndromes compared to psychiatric syndromes not associated with autoantibodies. Our cohort of autoantibody-associated psychiatric syndromes reveals a substantial overlap with possible and definitive autoimmune encephalitis. The risk in psychiatric patients to develop an autoimmune encephalitis if autoantibodies are present is about 9%. These data support our recently proposed model demonstrating that a mild brain inflammation might culminate later in autoimmune encephalitis [33]. However, note that as the ptau 181 neurodegeneration biomarker failed to differ significantly between our antibody-positive cohort and AD cohort, this evidence can be interpreted as an indication of axonal neurodegeneration in the antibody-positive group. Elevated ptau 181 in autoantibody-associated psychiatric disorders has been demonstrated [27,[34], [35], [36], [37]], and should be further validated in large-scale studies, as this is where diagnostic and therapeutic opportunities lie for psychiatric patients. Our finding that psycho-organic syndrome showed the most severe neurodegeneration among syndromes was expected because of its organic component and its overlap with symptoms of neurodegenerative dementia pathologies. Nevertheless, there should be more intensive research in this direction too, because the approach to infer a neurodegenerative component on the basis of psychiatric syndromes and accordingly, to improve the diagnostics and therapy of these diseases is so very promising.

## Funding

This study was funded by the Open Access fund of the 10.13039/501100003385University of Göttingen.

## Credit author statement

Conceptualization: ALJ and NH; Data curation, Formal analysis: ALJ; Investigation, Methodology, Project administration: ALJ and NH. Resources, Software: ALJ and NH. Supervision: all authors. Validation, Visualization, writing: ALJ and NH. All other authors revised the manuscript for important intellectual content.

## Declaration of competing interest

The authors declare that they have no known competing financial interests or personal relationships that could have appeared to influence the work reported in this paper.

## Data Availability

Data will be made available on request.
